# Bacteriopheophytin triplet state in *Rhodobacter sphaeroides* reaction centers

**DOI:** 10.1007/s11120-016-0290-6

**Published:** 2016-07-01

**Authors:** Rafał Białek, Gotard Burdziński, Michael R. Jones, Krzysztof Gibasiewicz

**Affiliations:** Faculty of Physics, Adam Mickiewicz University in Poznań, ul. Umultowska 85, 61-614 Poznan, Poland; School of Biochemistry, Medical Sciences Building, University of Bristol, University Walk, Bristol, BS8 1TD UK

**Keywords:** Carotenoid, Triplet, Bacteriopheophytin, *Rhodobacter sphaeroides*, Spheroidenone

## Abstract

It is well established that photoexcitation of *Rhodobacter sphaeroides* reaction centers (RC) with reduced quinone acceptors results in the formation of a triplet state localized on the primary electron donor P with a significant yield. The energy of this long-lived and therefore potentially damaging excited state is then efficiently quenched by energy transfer to the RC spheroidenone carotenoid, with its subsequent decay to the ground state by intersystem crossing. In this contribution, we present a detailed transient absorption study of triplet states in a set of mutated RCs characterized by different efficiencies of triplet formation that correlate with lifetimes of the initial charge-separated state P^+^H_A_^−^. On a microsecond time scale, two types of triplet state were detected: in addition to the well-known spheroidenone triplet state with a lifetime of ~4 μs, in some RCs we discovered a bacteriopheophytin triplet state with a lifetime of ~40 μs. As expected, the yield of the carotenoid triplet increased approximately linearly with the lifetime of P^+^H_A_^−^, reaching the value of 42 % for one of the mutants. However, surprisingly, the yield of the bacteriopheophytin triplet was the highest in RCs with the shortest P^+^H_A_^−^ lifetime and the smallest yield of carotenoid triplet. For these the estimated yield of bacteriopheophytin triplet was comparable with the yield of the carotenoid triplet, reaching a value of ~7 %. Possible mechanisms of formation of the bacteriopheophytin triplet state are discussed.

## Introduction

**Fig. 1:**
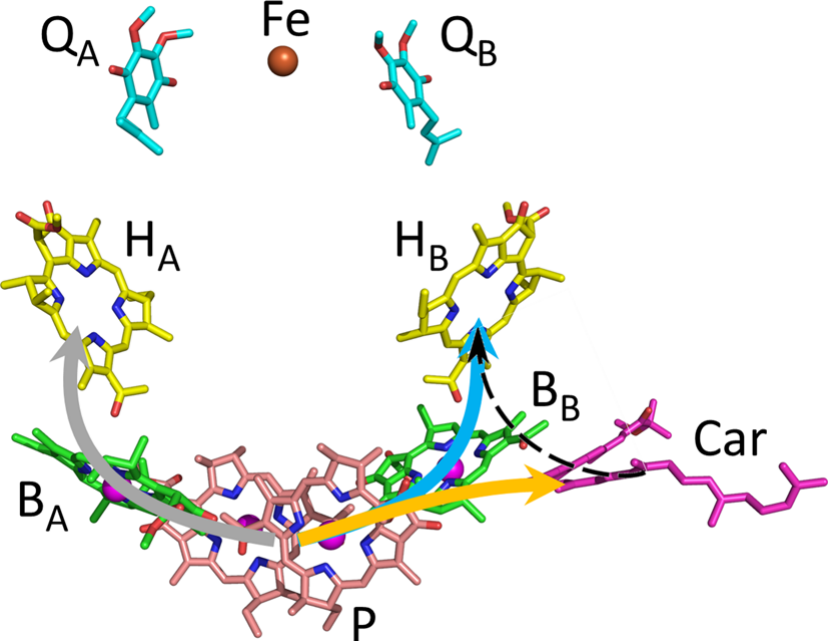
Routes of charge separation and triplet energy transfer in the *Rhodobacter sphaeroides* RC. The *gray arrow* shows the route of charge separation from P* to P^+^H_A_^−^ via P^+^B_A_^−^. The *orange* and *blue arrows* show the route of triplet energy transfer from ^3^P to the carotenoid and H_B_, respectively. The *dashed arrow* shows the possible route of triplet energy transfer from ^3^Car to ^3^BPhe. RC cofactors are shown with carbon atoms colored pink (P BChls), green (B_A_/B_B_ BChls), yellow (H_A_/H_B_ BPhes), magenta (carotenoid), and cyan (Q_A_/Q_B_ ubiquinones). Iron and magnesium atoms are shown as brown and magenta spheres, respectively

The primary photochemical reactions in photosynthesis take place in reaction center (RC) pigment-protein complexes. In the purple photosynthetic bacterium *Rhodobacter* (*Rba.*) *sphaeroides*, the RC comprises three polypeptide chains that provide a scaffold for four bacteriochlorophylls [BChls—two form a dimeric primary electron donor (P) and two are termed accessory BChls (B_A_ and B_B_)], two bacteriopheophytins (BPhes—H_A_ and H_B_), two ubiquinones (Q_A_ and Q_B_), a carotenoid (Car), and an iron ion (Allen et al. 1987; Yeates et al. 1988) (see Fig. 1). The carotenoid can be either spheroidene or spheroidenone depending on growth conditions, with mainly spheroidenone incorporated into the structure in the presence of oxygen (Schenck et al. 1984). The RC cofactors are arranged in two structurally pseudosymmetrical branches (A and B) differing in function. Branch A facilitates photochemical charge separation, while branch B plays a role in photoprotection by quenching the energy of any potentially harmful triplet states that may be formed during charge separation (Allen et al. 1987).

Electron transfer in the RC is initiated by formation of the first excited singlet state of the primary electron donor BChl dimer (P*), either by direct absorption of a photon or by energy transfer from any chromophore in the associated light harvesting pigment proteins (Stanley et al. 1996; Jordanides et al. 2001). Formation of P* triggers a charge separation in which the electron is transferred from P to H_A_ via B_A_ to form the state P^+^H_A_^−^. The route through which P^+^H_A_^−^ decays depends on whether the RC is “open,” with an oxidized Q_A_ ubiquinone acceptor, or “closed” with a reduced or absent Q_A_. In the laboratory, the RC can be closed by prereducing the Q_A_ ubiquinone, or removing it completely through chemical treatment or a gene mutation that changes the protein structure. In the open RC, the electron can be transferred onto Q_A_ and then Q_B_. In a closed RC, the state P^+^H_A_^−^ recombines either to the ground state (P) or to the triplet excited state (^3^P) via an intermediate triplet radical pair state ^3^(P^+^H_A_^−^) (Michel-Beyerle et al. 1979; Woodbury and Allen 1995). The ^3^P state is sufficiently energetic and long-lived (tens of microseconds lifetime) to sensitize singlet oxygen, producing photodamage, and is therefore an undesired product of charge separation. Any imbalance in the rates with which electrons leave the RC via the quinones and enter the RC via P^+^ has the potential to cause photodamage through the production of BChl triplet states and singlet oxygen (Cogdell et al. 2000).

The *Rba. sphaeroides* RC contains a Car molecule located next to the B_B_ (BChl), the principal function of which is to quench ^3^P (Frank and Cogdell 1996; Cogdell et al. 2000). The reported lifetime of the state ^3^P in the absence of carotenoid is 10–100 µs (Cogdell et al. 1975; Shuvalov and Parson 1981; Cogdell and Frank 1987; Frank and Violette 1989; Farhoosh et al. 1997; Arellano et al. 2004). The triplet energy is transferred from ^3^P to Car via a thermally activated pathway through the intervening B_B_, this being necessary because the triplet energy can be transferred only by the Dexter mechanism and Car is too distant from P for direct electron exchange (Frank and Violette 1989; Angerhofer et al. 1998; deWinter and Boxer 1999). The triplet state of the carotenoid (^3^Car) then decays via thermal deactivation without causing photodamage. The lifetime of ^3^Car in the *Rba. sphaeroides* RC is 2–10 µs for both spheroidene and spheroidenone (Monger et al. 1976; Schenck et al. 1984; Frank and Violette 1989; Frank et al. 1996; Farhoosh et al. 1997; Angerhofer et al. 1998; Arellano et al. 2004). In general, the precise triplet lifetime depends on the number of conjugated π bonds in the Car—the larger the number the shorter the lifetime (Kakitani et al. 2007). This number is 10 and 11 for spheroidene and spheroidenone, respectively.

Although, in nature, the toxic effect of triplet states is generally minimized through photoprotective mechanisms which prevent photodamage, the long-lived character of ^3^P has attractions for biotechnological applications in which the RC is used as a photosensitizer. As an example, Lukashev and coworkers (Lukashev et al. 2007) speculated that in a system with *Rba. sphaeroides* RCs immobilized on a TiO_2_ substrate, photosensitization occurs by injection of electrons from the RC ^3^P triplet state to TiO_2_. For such a technology, it could be desirable to increase the yield of ^3^P by manipulation of the structure and cofactor composition of the RC. For this reason, systematic characterization of triplet state pathways and target molecules in a range of engineered RCs with a variety of yields of triplet formation may help in finding a RC that is optimal for photovoltaic applications.

So far, the yield and mechanism of quenching of triplet states have been investigated in a variety of RCs with mutations near the A- and/or B-branch cofactors (Laible et al. 1998; deWinter and Boxer 1999; Marchanka et al. 2007; Gibasiewicz et al. 2011). A previous study by Gibasiewicz and coworkers (Gibasiewicz et al. 2011) explored the influence of point mutations around the cofactors of the A-branch on the yield of triplet formation, but did not address the localization of the triplet states or their lifetimes. The yield of triplet formation was found to decrease as the lifetime of the P^+^H_A_^−^ state became shorter.

In this study, apart from the triplet state of Car, another triplet state assigned to BPhe was detected and characterized by microsecond transient absorption spectroscopy in a range of mutated membrane-embedded and wild-type (WT) detergent-purified *Rba. sphaeroides* RCs. In all cases, a spheroidenone triplet state was formed and decayed with similar lifetimes of ~4 µs. The yield of ^3^Car was strongly modulated ranging from 4 to 47 % in different samples. An additional long-lived triplet state assigned to BPhe (^3^BPhe) had a lifetime of ~40 μs and yield of up to 5 %. Importantly, the efficiency of formation of the ^3^BPhe state was inversely correlated with the efficiency of formation of the ^3^Car state.

## Materials and methods

RC-enriched membranes from antenna-deficient strains of *Rba. sphaeroides* and purified RCs were prepared as described previously (Gibasiewicz et al. 2011). The set of point mutations employed in this study were also described in detail recently (Gibasiewicz et al. 2011). In brief, the following mutants were studied: AMW (Ala M260 replaced by Trp), YMF (Tyr M210 by Phe), YMW (Tyr M210 by Trp), GML (Gly M203 by Leu), YLH (Tyr L128 by His), FLY (Phe L146 by Tyr), FLA (Phe L146 by Ala) and ELL (Glu L104 Leu). Apart from RC-membranes, four preparations of WT- and ELL-purified RCs were used (described below). Throughout the text, abbreviations of the samples names with extension “-RC” denote purified RCs, whereas those without this extension denote RC-enriched membranes.

Prior to measurements, stock solutions of RC-membranes and purified RCs were diluted with buffer to an absorbance of 0.7 (±5 %) at 760 nm in 1-cm cuvette. Buffer for RC-membranes was 20 mM Tris–HCl (pH 8.0) and for purified RCs the same with an addition of 0.1 % LDAO (lauryldimethylamine-*N*-oxide; Sigma-Aldrich). Sodium ascorbate and o-phenanthroline were then added to final concentrations of 10 mM both to all RC-membranes and two samples of purified RCs (WT-RCoph and ELL-RCoph). Sodium ascorbate reduced P^+^ to P (the samples were continuously illuminated with weak background white light forming the state P^+^Q_A_^−^), thus leaving all the RCs in the closed state with Q_A_^−^. Addition of o-phenanthroline replaced the ubiquinone in the Q_B_ binding site thus blocking electron transfer from Q_A_^−^ to Q_B_ (Gibasiewicz et al. 2011). The o-phenanthroline was prepared as a 250 mM stock solution in ethanol yielding final concentration of ethanol in the sample of 4 %. Additional samples of purified RCs (WT-RC and ELL-RC) were prepared with 10 mM sodium ascorbate and 4 % ethanol with no o-phenanthroline added. Purified RCs are partially devoid of quinone Q_B_ and thus o-phenanthroline was not necessary to keep the fraction of RCs without quinone Q_B_ closed.

The transient absorption measurement system was constructed as described previously (Burdzinski et al. 2011). Briefly, pump pulses (532 nm; 8 ns FWHM) were generated at a repetition rate of 0.5 Hz by a Q-switched Nd:YAG laser (Continuum Surelite II). For the probe light, a 150-W xenon arc lamp (Applied Photophysics) was used in pulsed mode with a repetition rate of 1 Hz. A monochromator (Acton Research Spectra Pro 300i) was used to disperse the probe light which was then detected by photomultiplier (R928 Hamamatsu) connected to a digital oscilloscope (Tektronix TDS 680 C). Samples were placed in a quartz cuvette (1 × 1 cm cross section).

In most cases, transient absorption difference measurements were performed in the range of 435–850 nm for RC-membranes or 375–835 nm for purified WT-RCs in 15-nm steps. The smaller spectral range used for RC-membranes was due to intensive light scattering at shorter wavelengths. Excitation was at 532 nm and the temporal window of the measurements was either 13.5 µs (GML, FLY, FLA, YLH), 27 µs (AMW, YMW, YMF, WT, WT-RC, WT-RCoph), 54 µs (ELL), or 540 µs (ELL-RC, ELL-RCoph) for all detection wavelengths. In a few individual cases (ELL, YMF, and purified WT-RCs), measurements at selected wavelengths were repeated over a longer time window (135 or 270 µs). The excitation energy per pulse was in the range of 0.1–1.1 mJ, and the data were typically normalized in respect to the excitation energy used for each sample. All kinetics were results of averaging of 30 traces.

Measurements were conducted under ambient atmosphere apart from a few in an argon atmosphere. The procedure of deoxygenation of samples was as follows. Samples were placed in a sealed cuvette and a stream of argon was directed onto the surface of solution instead of bubbling into the solution in order to prevent formation of foam. Argon treatment lasted 20 min and was performed just before measurement. The efficiency of the described procedure was tested with the aqueous solution of phenalenone, which is a well-known oxygen sensitizer (Schmidt et al. 1994). The deoxygenation extended the phenalenone triplet lifetime from 1.7 to 80 µs.

For each sample, steady-state absorption spectra were taken before and after the transient absorption measurements. Due to large size of RC-membranes, their spectra were distorted by light scattering particularly contributing at shorter wavelengths. After each transient absorption experiment, the lineshape of the RC-membranes absorption spectra were not significantly changed but for some samples, and background light scattering was increased somewhat. The small gradual loss of transparency of these samples during the transient absorption measurements was monitored during the experiment as a loss of the signal at 600 nm and was corrected for.

Transient absorption kinetic traces were typically fitted with the sum of two exponentials with or without an offset using the global analysis algorithm in program Asufit (http://www.public.asu.edu/~laserweb/asufit/asufit.html) from Arizona State University and OriginPro. The starting point of fit was at the maximum of the transient signal at about 150 ns.

## Results

### Kinetics of decay

Transient absorption kinetics were measured for membrane-embedded WT-RCs, a range of membrane-embedded engineered RCs and detergent-purified WT- and ELL-RCs (see “Materials and methods” section). RCs were treated with sodium ascorbate and weak background illumination to reduce Q_A_, blocking charge separation at the radical pair P^+^H_A_^−^. With one exception the engineered RCs contained a single amino acid change that either introduced or removed a hydrogen bond or charge-dipole interaction with H_A_, B_A_ or the P BChls. As described in detail elsewhere (Gibasiewicz et al. 2011), these changes modify the free energies of the states P^+^B_A_^−^ and/or P^+^H_A_^−^ in a variety of ways, with resulting modifications in the rate of charge separation and charge recombination. The exception was the AMW engineered RC which lacks a Q_A_ ubiquinone such that charge separation is blocked at P^+^H_A_^−^ without the need for treatment with ascorbate and background illumination.

**Fig. 2:**
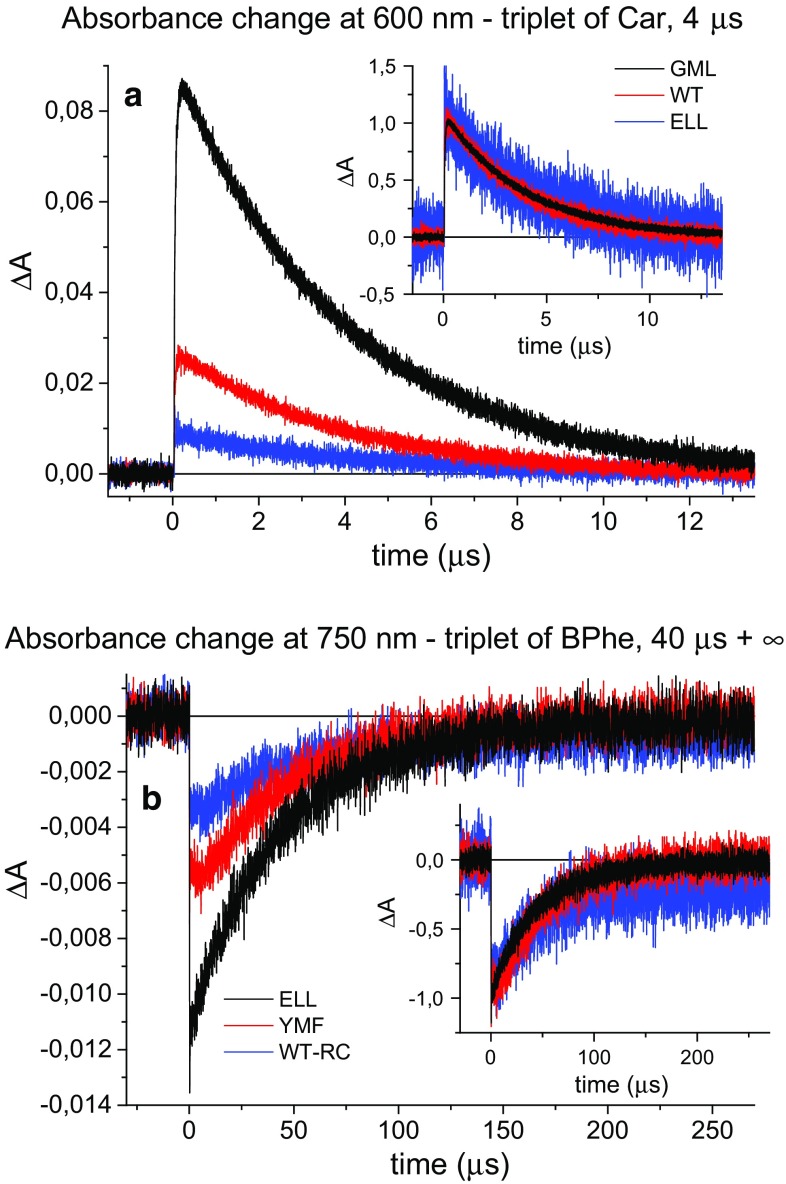
Absorbance changes for selected RCs measured at 600 and 750 nm after excitation at 532 nm. **a** The kinetics reveal decay of the triplet state of spheroidenone (Car). **b** The kinetics reveal decay of the triplet state of BPhe. In the main figures, the kinetics were normalized to the same excitation energy and sample OD at ~760 nm. In the insets, the kinetics were normalized to the same initial amplitude. Data in (**b**) were smoothed using a Savitzky–Golay algorithm with a 10-points window

Kinetics were measured across a range of wavelengths following photoexcitation at 532 nm (see “Materials and methods” section). The absorption of the triplet state of spheroidenone is expected to exhibit maximum at ~600 nm (Arellano et al. 2004), and a few typical kinetic traces measured at this wavelength are shown in Fig. 2a. The major difference between the three traces was their initial amplitude, reflecting differences in the formation yield of the carotenoid triplet state. Following the fast increase of the signal, a ~4 μs component (3.8–4.5 µs for different RCs see Table 1, *τ*_1_) dominating the overall decay was observed in all RCs (see normalized traces in inset of Fig. 2a).

**Table 1:** Mean lifetimes of P^+^H_A_^−^ and lifetimes and yields of triplet states in RC-membranes and purified RCs

Sample	$\tau_{\text{PH}} \left( {\text{ns}} \right)$ (Gibasiewicz et al. 2011)	$\tau_{1} \left( {\mu {\text{s}}} \right)$	*Φ*_T_	%_Car_	%_BPhe_	*Φ*_TCar_	*Φ*_TBPhe_
AMW	17.0	3.9	0.25	98	2	0.25	0.005
YMF	11.6	4.0	0.21	82	18	0.17	0.037
GML	11.5	3.9	0.41	96	4	0.39	0.018
WT	7.7	4.0	0.14	92	8	0.13	0.010
YMW	7.5	3.9	0.22	90	10	0.19	0.022
YLH	5.4	4.0	0.13	82	18	0.10	0.024
FLY	3.0	4.0	0.05	65	35	0.04	0.019
FLA	2.7	4.3	0.10	57	43	0.06	0.044
ELL	2.2	4.5	0.11	39	61	0.04	0.069
WT-RCoph	–	3.9	0.28	86	14	0.24	0.039
WT-RC	–	3.8	0.15	75	25	0.11	0.037
ELL-RCoph	–	4.1	0.21	69	31	0.14	0.066
ELL-RC	–	4.0	0.08	57	43	0.04	0.034

$\varvec{ \tau }_{\text{PH}} \varvec{ }$ mean lifetime of the state P^+^H_A_^**−**^, ${\tau}_{1}$ lifetime of triplet state of Car obtained from biexponential global analysis, ${{\Phi}}_{\text{T}}$ total triplet formation yield, ${\% }_{\text{Car}} ,{\% }_{\text{BPhe}}$ percentage contributions of triplet state on Car and BPhe, ${{\Phi}}_{\text{TCar}} ,\,{{\Phi}}_{\text{TBPhe}}$ yields of triplet formation on Car and BPhe. ${\tau}_{2}$, the lifetime of triplet state of BPhe, was fixed at 40 μs. A possible minor contribution of the triplet state localized on bacteriochlorophyll(s) in some samples was neglected in the calculations

For most RCs, an additional longer decay component was also observed. In most cases, its amplitude contribution was the highest at a probe wavelength 750 nm and the decays at this wavelength is shown in Fig. 2b (traces normalized to initial amplitude are also shown in the inset). In addition, at 750 nm there was a small raising phase of the negative signal on a time scale of the first few microseconds after excitation [most clearly visible for the YMF RC-membranes in Fig. 2b (red)].

Most measurements were performed in a 13.5, 27, or 54 μs time window (see “Materials and methods” section), but for some samples and wavelengths a longer time window (135, 270 or 540 μs) was used (purified ELL-RCs—whole spectrum; ELL, YMF membranes and purified WT-RCs—selected wavelengths). These experiments allowed a time constant of 30–60 μs (with an average of 40 μs) for the longer decay component to be determined. A lifetime value of 40 μs was kept fixed in biexponential fitting of decays for all samples to account for this component. In addition, for purified WT- and ELL-RCs an additional nondecaying component was observed.

### Absorbance difference spectra of purified RCs

**Fig. 3:**
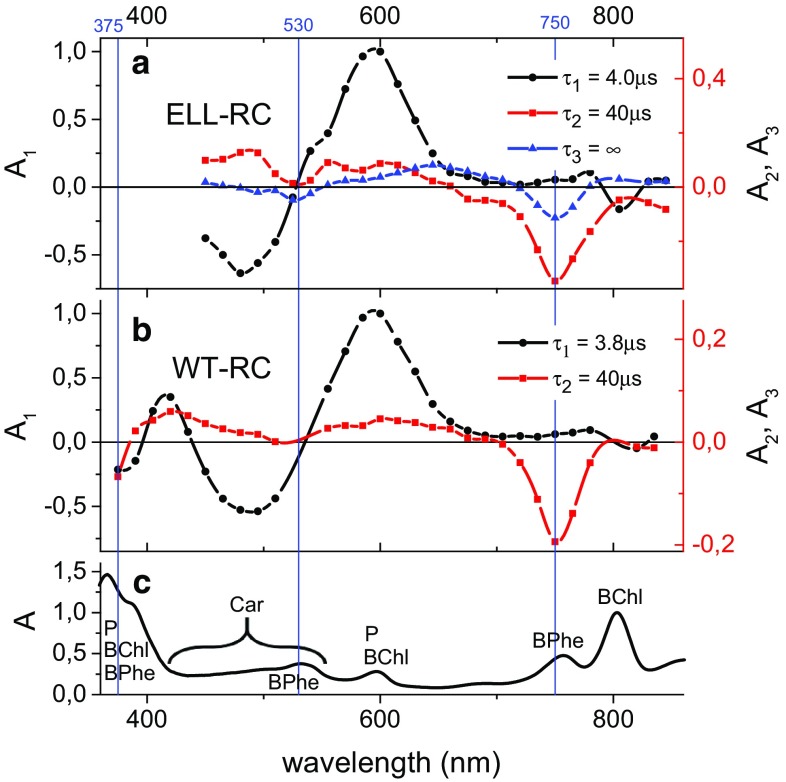
Comparison of decay-associated spectra for WT- and ELL-purified RCs without o-phenanthroline and the steady-state absorption spectrum of WT-RCs. **a** Fit for ELL-RCs over a 540-μs time window. **b** Fit for WT-RCs over a 27-μs time window. **c** Absorption bands labeled according to the relevant RC cofactors. The DAS were obtained from a global analysis using the fitting function Δ*A*(*λ*) = *A*_1_(*λ*)exp(−*t*/*τ*_1_) + *A*_2_(*λ*)exp(−*t*/*τ*_2_) + *A*_3_ (with *A*_3_ = 0 for WT-RC). Note the different scales of the ordinate axis for the DAS of the slow components (*right axis*)

Measurements of decay kinetics at a range of wavelengths allowed the construction of transient absorption difference spectra which were then analyzed using a global fitting procedure (van Stokkum et al. 2004) to yield decay-associated spectra (DAS). The most complex spectral evolution was obtained with purified ELL-RCs which had not been treated with o-phenanthroline (ELL-RC). Data for these RCs were best fitted with two exponentials and a nondecaying component (offset), addition of a third exponential component not improving the fit significantly. Figure 3a presents the DAS for the three components of the decay in ELL-RC. The absorbance changes were measured every 40 ns over a time window of 540 μs. As can be seen, the whole spectrum was dominated by the shortest (~4-µs) component except for the region at around 750 nm, corresponding to the BPhe absorbance band (Fig. 3c) where the other two components had a higher amplitude. Data for the WT-RC collected over a time window of 27 µs (Fig. 3b) could be fitted with two exponentials, but addition of an offset resulted in an additional featureless DAS and did not improve the fit significantly. The lineshapes of the DAS for the WT-RC were similar to those for the ELL-RC.

### Absorbance difference spectra of all RC samples

**Fig. 4:**
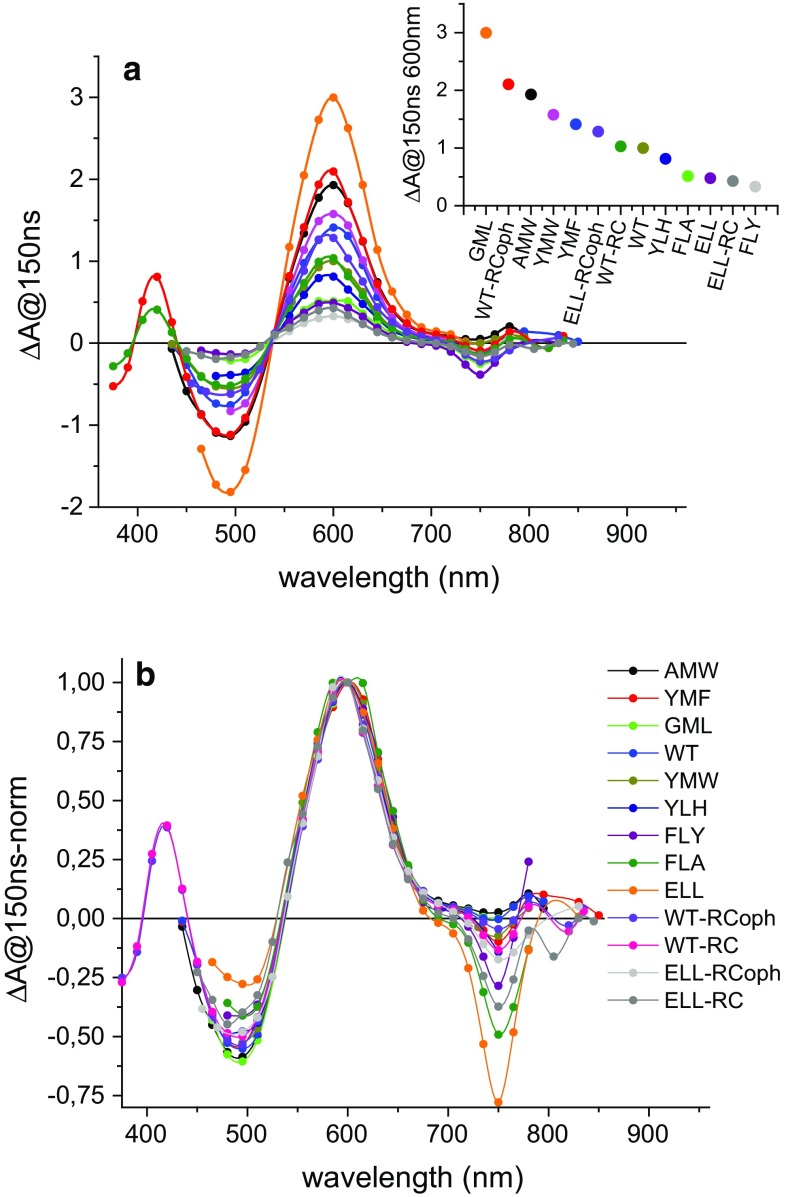
Comparison of the initial transient absorption spectrum for all RC-membranes and purified RCs (with and without o-phenanthroline) excited at 532 nm. The spectra were constructed from the values of absorbance changes measured 150 ns after excitation (at the signal maximum, ~600 nm). **a** Spectra normalized to the same excitation energy and sample OD at ~760 nm. The absorbance change for WT RC-membranes was arbitrarily set to 1 at 600 nm. The *inset* shows a comparison of maximum amplitudes of the spectra at 600 nm relative to WT. **b** Transient absorption spectra normalized to the same amplitude at 600 nm. The order of samples in the legend in (**b**) (and also in Figs. 5, 6) is from longest to shortest lifetimes of P^+^H_A_^−^ for RC-membranes, with purified RCs added at the end

Figure 4a presents absorbance difference spectra at 150 ns delay for all the RCs investigated. Data for purified RCs were collected across a broader spectral range than for RC-membranes, as they were less scattering at shorter wavelengths, allowing observation of an additional positive band at 420 nm. As can be seen from Fig. 4b, where the spectra were normalized to the peak at 600 nm, all spectra were similar in lineshape apart from the depth of the negative features at 495 and 750 nm. The most distinctive were the spectra of the ELL and FLA RC-membranes which had the deepest ~750 nm trough and the shallowest ~495 nm trough.

All data were treated with the global analysis algorithm described above for the purified RCs. Only data for purified ELL-RCs (with and without o-phenanthroline) required fitting with two exponentials and an offset. For all other membrane-bound RCs and purified WT-RCs, a two exponential model without an offset was satisfactory.

**Fig. 5:**
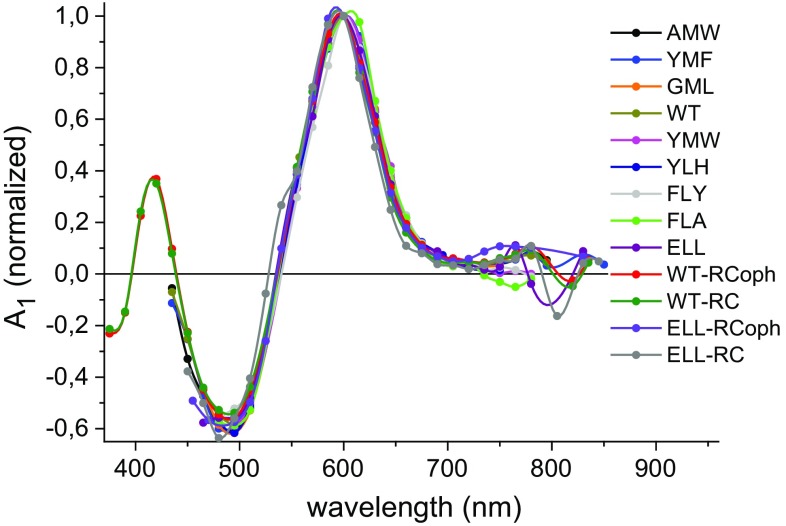
Decay-associated spectra of the τ_1_ = ~ 4 µs component. The DAS were obtained from a global analysis using the fitting function Δ*A*(*λ*) = *A*_1_(*λ*)exp(−*t*/*τ*_1_) + *A*_2_(*λ*)exp(−*t*/*τ*_2_), except for ELL-RC and ELL-RCoph for which additional nondecaying component (*A*_3_) was added. Spectra were normalized by dividing them by the value of *A*_1_ (600 nm) for each sample. Fits were performed in 13.5 μs time window for GML, FLY, FLA, and YLH, 54 µs for ELL, 540 µs for ELL-RC and ELL-RCoph, and 27 µs for the remaining

**Fig. 6:**
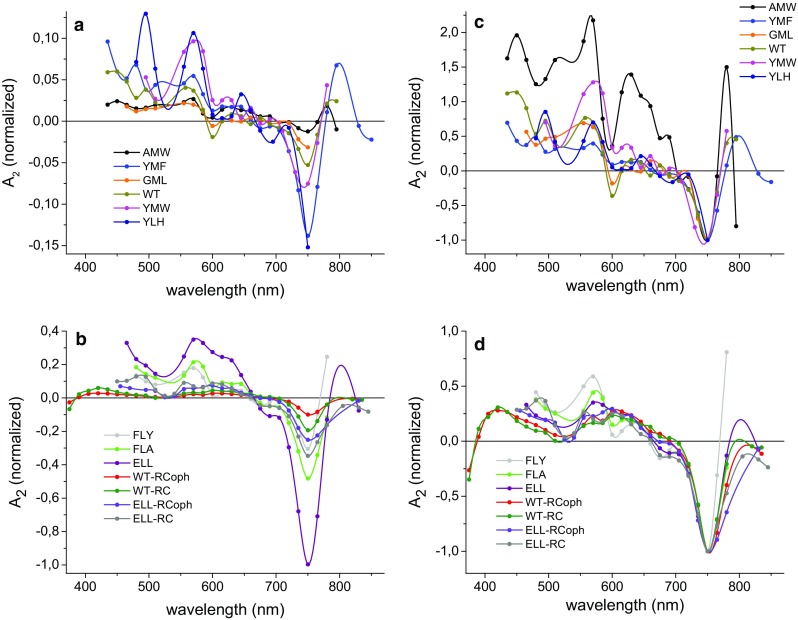
Decay-associated spectra of the *τ*_2_ = ~40 µs component. The DAS were obtained from global analysis using the fitting function Δ*A*(*λ*) = *A*_1_(*λ*)exp(−*t*/*τ*_1_) + *A*_2_(*λ*)exp(−*t*/*τ*_2_) except for ELL-RC and ELL-RCoph for which additional nondecaying component (*A*_3_) was added. Spectra in **a** and **b** are normalized by dividing by the value of *A*_1_ (600 nm) for each sample. Note that the scale of the ordinate axis is ~4.5-fold larger in panel b than in panel a. Spectra in **c** and **d** are normalized to a value of −1 value at 750 nm. Fits were performed in the same time windows as in Fig. 5

DAS of the fast (*τ*_1_) and slow (*τ*_2_) lifetime components of each two-exponential fit are presented in Figs. 5, 6, respectively. The DAS of the fast component were almost the same in shape for all RCs, with a dominating positive signal at ~600 nm, a small positive signal at ~750 nm, and a negative signal at ~495 nm. The positive value of this DAS around 750 nm corresponded well with the shape of the kinetics in Fig. 2b, which showed a short increasing phase of the negative signals for YMF and WT-RC. The major difference between the spectra in Fig. 5 was a difference in overall amplitude before normalization.

The DAS of the slow component (Fig. 6) showed a clear minimum at ~750 nm, a broad and essentially positive structure between ~660 and ~400 nm (Fig. 6a, b), and the red edge at ~395 nm of a negative band for purified WT-RCs (Fig. 6b, d). The lineshape of this slow component DAS was not identical among all RCs (Fig. 6c, d), with peaks positions being approximately the same but the relative amplitude of these peaks and detailed structure differing between RCs. The most strongly diverging DAS was that for the AMW RC-membranes for which the absolute negative amplitude at 750 nm was the smallest.

**Fig. 7:**
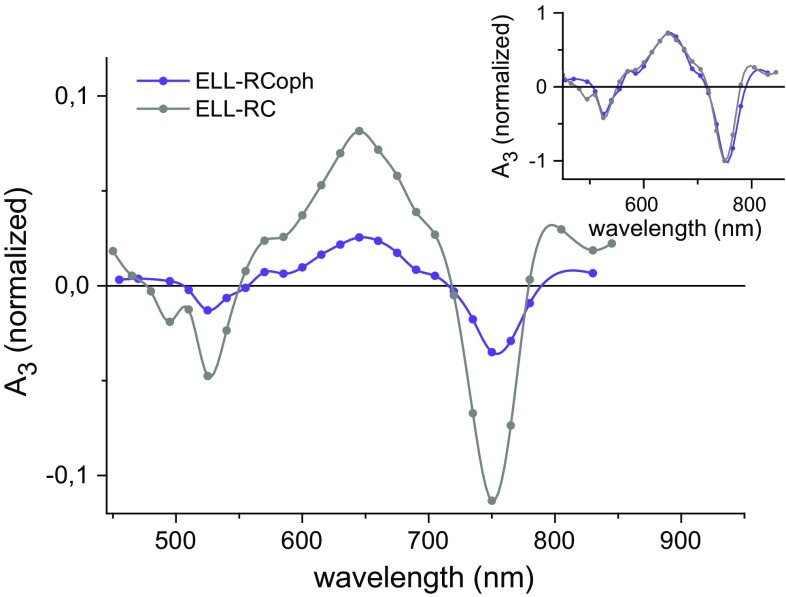
Decay-associated spectra of the nondecaying component for purified ELL-RCs. The DAS were obtained from a global analysis using the fitting function Δ*A*(*λ*) = *A*_1_(*λ*)exp(−*t*/*τ*_1_) + *A*_2_(*λ*)exp(−*t*/*τ*_2_) + *A*_3_. Spectra in the main figure were normalized by dividing them by the value of *A*_1_(600 nm) for each sample. The spectra in the inset are normalized to a value of −1 at 750 nm. Fits were performed over a 540-μs time window

The DAS of the nondecaying component (Fig. 7) required for fits to data on purified ELL-RCs was similar in shape to the slow component DAS apart from the structure of the positive band, which was red shifted in the nondecaying component. The high quality of the spectra of this component was due to the broad time window (540 µs). The two DAS obtained clearly showed a maximum at around 650 nm and two minima at around 750 and 530 nm, and differed from one another mainly in terms of amplitude (see normalized spectra in the inset of Fig. 7).

### The influence of oxygen

Kinetics at 600 and 750 nm for samples of ELL RC-membranes and ELL-purified RCs without the addition of o-phenanthroline (ELL-RC) were obtained after argon treatment as described in Materials and methods. These kinetics (data not shown) did not show any difference compared to kinetics measured in an ambient atmosphere.

## Discussion

The measurements described above examined the spectral properties of long-lived states formed after photoexcitation of membrane-bound or purified RCs in which forward electron transfer from H_A_^−^ to Q_A_ was blocked either by application of ascorbate plus illumination or by a genetic change in the AMW RC. As recombination of P^+^H_A_^−^ occurs in a few tens of nanoseconds, light-induced absorbance changes persisting on a microsecond time scale are attributable to long-lived triplet states. Two such states were detected in data collected using a range of RCs that are known to show varying rates of recombination of P^+^H_A_^−^ and, in those with the slowest recombination, an increased yield of triplet states (Gibasiewicz et al. 2011).

### Attribution of the fast decay component to a Car triplet state

The DAS of the ~4 μs decay component (Fig. 5) can be ascribed to the triplet state of spheroidenone (Schenck et al. 1984; Arellano et al. 2004), the trough at ~495 nm corresponding well to the maximum of the broad steady-state absorption band of the single spheroidenone in the RC (Fig. 3c). The lifetime of ~4 µs is consistent with published values for the lifetime of the spheroidenone triplet state in purple bacterial pigment-protein complexes (RCs, LH1, and LH2) of around 4–5 µs (Schenck et al. 1984; Arellano et al. 2004; Kakitani et al. 2007).

### Identification of the slow decay component as a BPhe triplet state

In most RCs, the most prominent feature of the DAS of the slow, ~40 µs decay component was a trough at ~750 nm (Figs. 3a, b, 6). As can be seen from Fig. 3c, this corresponded well to the lowest energy absorption band of the two RC BPhes at 756 nm, suggesting that this component may be attributed to the triplet state of one or both of the RC BPhes. The shape of this component resembled the BPhe triplet absorbance difference spectrum acquired in methanol-acetone solution (Holten et al. 1976), and lifetimes of a BPhe triplet state in methanol-acetone, ethanol, or toluene solutions have been reported to vary between 16 and 30 µs (Holten et al. 1976; Yang et al. 2011) in good agreement with the average ~40 µs time constant observed in the present work. To our knowledge, the triplet spectrum of one or both of the BPhes in the RC protein has not been reported previously. However, a contribution from photobleaching of the accessory BChls or P, revealing the triplet states of these molecules, to the carotenoid triplet spectra has been observed (Lous and Hoff 1989; Frank and Violette 1989; Angerhofer et al. 1998; Arellano et al. 2004) and attributed to a mixed population of RCs with and without Car. No signature of the triplet state of the accessory BChls was observed in the Q_y_ region in the present work (i.e., there was a lack of any photobleaching at 800 nm in the 40 μs DAS, with the possible exception of data recorded for AMW RC-membranes—Fig. 6c), although a small trough at ~600 nm (corresponding to the Q_x_ bands of BChl and P) was present in the DAS for some RC-membranes (Fig. 6a, b) indicating a possibility of a small admixture of the triplet state of P. As an alternative attribution, the bleaching at 750 nm could be ascribed to H_A_^−^ in a ^3^[P^+^H_A_^−^] state that is in an equilibrium with a ^3^P state, but this possibility is challenged by the lack of a significant photobleaching in the ~820–850 nm region (Fig. 6) where the short-wavelength tail of P Q_y_ photobleaching signal would be expected. The spectra of the slow component were not identical in shape among all the RC samples (Fig. 6c, d). This may be due to the above-mentioned admixing of different states in different contributions (^3^BChl or ^3^P) to the ^3^BPhe triplet state, or the influence of point mutations on the shape of the ^3^BPhe spectrum. The possibility of ^3^BPhe state formation has previously been suggested on the basis that singlet oxygen formation was found to be higher in thermally treated RCs with induced pheophytinization of BChls (Uchoa et al. 2008).

### Identification of the nondecaying component

Photobleaching bands in the DAS of the nondecaying component at 530 and 750 nm corresponded well with the absorption bands of BPhe in the steady-state spectrum, suggesting that this component could also be ascribed to the triplet state of one or both BPhes. This component was required for fitting only for purified ELL-RCs and, comparing the lineshapes of the slow component DAS for WT-RCs (Fig. 3b, red) and the slow and nondecaying components for ELL-RCs (Fig. 3a, red and blue), it seems plausible that the single DAS for the WT-RC is equivalent to the sum of the two DAS for the ELL-RC. This statement is supported by the presence of a nondecaying component in the WT-RC kinetics in Fig. 2b. By comparing spectra of the nondecaying component (Fig. 3a, 7) with those of the 40 µs component (Fig. 3a, b), it can be seen that there are differences in the positive band below 700 nm, this band being red shifted in the spectra of the nondecaying component. The origin of this could be heterogeneity of sample or the protein dynamics on the microsecond time scale.

### Effect of deoxygenation

As it was described in Results, there is no influence of deoxygenation on the lifetime of the triplet state of both BPhe and Car. The role of spheroidenone is to prevent RCs from damage from singlet oxygen by quenching the triplet state of P (Cogdell et al. 2000) so it is not surprising that the oxygen does not quench ^3^Car. On the other hand, it is reported in the literature that BPhe should sensitize singlet oxygen (Uchoa et al. 2008). The likely reason why this sensitization was not observed in described experiments is the presence of ascorbate, which was reported to be triplet suppressor. Concentration of the order of 50 µM of ascorbate can decrease singlet oxygen formation by 20 % (Uchoa et al. 2008), so 10 mM concentration could suppress it totally.

### Relative contributions of the two triplet states and their absolute yields

Neglecting a possible minor contribution from ^3^P and/or ^3^BChl as discussed above, the molar fractions of the triplet states located on BPhe and Car were estimated by assuming that the amplitude of the faster decay at 495 nm came only from photobleaching of Car and the sum of the amplitudes of the two components at 750 nm came from photobleaching of BPhe. Thus, the percentage contribution of each triplet state was determined from the following formula (see “Appendix” for full derivation):

1 $$\%_{\text{Car}} = \frac{{1.09 \times A_{{1;495\,{\text{nm}}}} }}{{A_{{1;750\,{\text{nm}}}} + A_{{2;750\,{\text{nm}}}} + 1.09 \times A_{{1;495\,{\text{nm}}}} }} \times 100\,\%$$

2 $$\%_{\text{BPhe}} \, = \, 100\,\% - \%_{\text{Car}}.$$

The resulting percentage populations are presented in Table 1, and ranged from ~98 %/2 % to 39 %/61 % for ^3^Car/^3^BPhe.

On the assumption that $A_{{1;495\,{\text{nm}}}}$ is a specific measure of the ^3^Car formed, one can also calculate the total yield of triplet (*φ*_T_) in arbitrary units to compare between samples:

3 $$\varPhi_{\text{T}} = \frac{{A_{{1;495\, {\text{nm}}}} }}{{\%_{\text{Car}} }} \times 100\,\%.$$

It has been reported that in Q_A_-reduced WT-RCs, the yield of triplet formation is 0.15 (Parson and Cogdell 1975; Michel-Beyerle et al. 1980; Schenck et al. 1982). This value was obtained for purified RCs reduced with sodium dithionite, which is most similar to our purified WT-RC sample with sodium ascorbate and without o-phenanthroline. Thus, a value of 0.15 was ascribed to the triplet yield for this preparation of WT-RC and all other values were calculated relative to this. The resulting total triplet formation yields are presented in Table 1 along with absolute contributions from ^3^Car and ^3^BPhe. One can see that almost the same value of total triplet yield was obtained for WT RC-membranes (0.14) as for purified WT-RC (0.15). Mutation AMW leads to genetic depletion of Q_A_ in RC, and the triplet formation yield obtained for this RC (0.25) corresponded well with a value of 0.32 ± 0.04 obtained for purified WT-RCs with chemical depletion of Q_A_ (Chidsey et al. 1984). This observation reinforces our confidence in the correctness of the calculation algorithm.

### Mutations revealed a correlation between P^+^H_A_^−^ lifetime and triplet yields

**Fig. 8:**
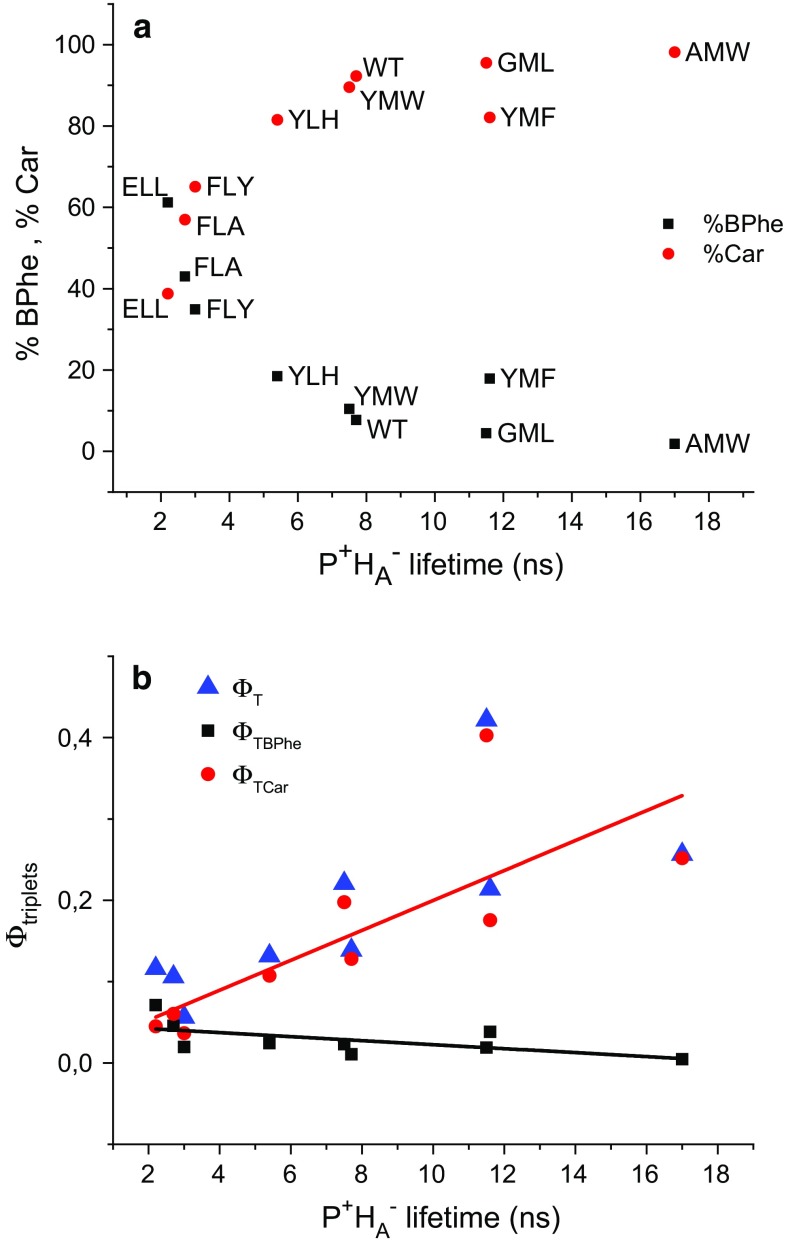
Dependence of triplet formation efficiencies on the mean lifetime of P^+^H_A_^−^ in RC-membranes. **a** Relative contribution of triplet states on BPhe and car. **b** Absolute triplet formation yields on BPhe (*Φ*_TBPhe_), Car (*Φ*_TCar_), and their sum (*Φ*_T_). The color lines indicate trends in the changes of *Φ*_TBPhe_ and *Φ*_TCar_ vs. P^+^H_A_^−^ lifetime

A factor which could affect the yield of triplet states in a mutated RC is the impact of the mutation on the mean lifetime of P^+^H_A_^−^ state. This was determined in previous work on this set of mutated membrane-embedded RCs (Gibasiewicz et al. 2011) and the mean lifetimes obtained are presented in Table 1. The plots in Figs. 8a, b present the dependence of the relative yields of ^3^Car and ^3^BPhe, and absolute yields of triplet, respectively, on the mean lifetime of P^+^H_A_^−^. As can be seen from Fig. 8b, the total triplet yield increased with increasing P^+^H_A_^−^ lifetime, and this was due to an increase in the yield of carotenoid triplet. Although the absolute level of ^3^BPhe formation did not vary greatly (Fig. 8b), its relative contribution was particularly strong in those mutants that had a strongly accelerated rate of P^+^H_A_^−^ decay (Fig. 8a). This may be caused by point mutations affecting the energy levels of chromophores in branch A.

### Effect of o-phenanthroline on triplet yields in purified RCs

The yield of total triplet formation in purified WT-RCs with o-phenanthroline (WT-RCoph—0.28) was similar to that in membranes mutant RCs lacking Q_A_ (AMW—0.25). It was reported (Gibasiewicz and Pajzderska 2008) that o-phenanthroline slows down P^+^H_A_^−^ recombination by screening the negative charge on Q_A_^−^ that makes this RC similar to the AMW RCs and explains the similarity between these two. On the other hand, the total triplet yields of both WT- and ELL-purified RCs without o-phenanthroline were significantly lower than the respective yields for purified RCs with o-phenanthroline. This effect may be caused by (a) faster P^+^H_A_^−^ recombination in the absence of o-phenanthroline and/or (b) presence of a significant fraction of RCs in open state, i.e., containing quinone Q_B_ that accepts electrons from Q_A_^−^, RCs being unable to form triplet states due to very short lifetime of P^+^H_A_^-^ in purified RCs without o-phenanthroline. Both for WT- and ELL-purified RCs, the positive influence of o-phenanthroline on the Car triplet yield is qualitatively the same as the effect of this compound on the total triplet yield (Table 1). In the case of purified ELL-RCs, also BPhe triplet yield increases after addition of o-phenanthroline, although the effect is less spectacular. Oppositely, the BPhe triplet yield for purified WT-RCs is almost unchanged after addition of o-phenanthroline. These observations suggest that in purified RCs there is no clear positive correlation between the lifetimes of P^+^H_A_^−^ and BPhe triplet yields, similarly as it was shown for RC-enriched membranes (Fig. 6b).

### Possible mechanisms for the formation of ^3^BPhe

There are at least three possible explanations for the formation of a ^3^BPhe state: (1) the transfer of a triplet excited state from primary donor to the H_B_ BPhe with some probability in all RCs, (2) formation of a triplet state of BPhe directly from its singlet state by intersystem crossing (ISC), bearing in mind that an excitation wavelength of 532 nm was used which corresponds to the overlapping Q_X_ bands of the two RC BPhes, or (3) the occurrence of a subpopulation of damaged RCs.

Regarding mechanism 1, it is well established that ^3^P is quenched by carotenoid through the intermediate formation of the triplet state of the accessory BChl B_B_ (see Introduction), a reaction that is thermally activated. One possibility therefore is that the energy of ^3^P is transferred with some probability from B_B_ to the H_B_ BPhe rather than to the Car. ^3^BPhe is almost not observed in RC-membranes containing WT-RCs and so probably energy levels are normally unfavorable for this triplet transfer. The fact that higher levels of ^3^BPhe triplet were seen in both samples of purified WT-RC could be explained by a shift in these energy levels that activates a low yield of triplet energy transfer from ^3^P to ^3^BPhe. In support of this, it is well known that charge separation in WT-RCs in RC-membranes is somewhat slower than in purified WT-RCs (P* lifetime of 5 ps versus 3–3.5 ps). It is less easy to explain why the mutations YMW, YLH, FLY, FLA, and ELL would also affect the yield of a H_B_ triplet in RC-membranes, as each of these mutations is in the vicinity of P or the A-branch B_A_ and H_A_. One possible explanation is that the observed triplet state is localized on BPhe H_A_ rather than H_B_. However, given the well-established mechanism (Angerhofer et al. 1998) that the triplet naturally goes via the B branch through B_B_ to the Car, this would seem to favor BPhe H_B_ as the final carrier of the triplet. Another possibility is triplet transfer from ^3^P to H_B_ through the Car. As it can be seen in Fig. 5, for most of the samples the fast component DAS was slightly positive at around 750 nm which could be the signature of transfer of triplet state energy from the Car to BPhe. The same process is also seen in Fig. 2b as a small raising phase of the negative signals in YMF and WT-RC kinetics. This route would also favor H_B_ as the triplet acceptor as it is much closer to Car than H_A_. However, this positive signal could be also a tail of triplet–triplet absorption of Car so this requires further investigation.

Regarding mechanism 2, it has been proposed in the literature that a triplet state may be formed on BPhe by intersystem crossing (Uchoa et al. 2008), but on the other hand results from EPR spectroscopy have been presented that show that ^3^BPhe formation via intersystem crossing is unlikely (Marchanka et al. 2007). Although in the present study the RCs were excited at 532 nm, implying initial formation of the singlet excited states of H_A_/H_B_, expectations from the well-characterized mechanism of RC energy transduction, is that excited state energy should be passed on a subpicosecond time scale to P* (Stanley et al. 1996; Jordanides et al. 2001), or that charge separation should be initiated from an alternative state such as B_A_* (van Brederode et al. 1997). Given this, it seems unlikely that a persistent ^3^BPhe state with a lifetime of tens of microseconds could be explained by direct formation of H_A_* or H_B_* after 532 nm excitation followed by intersystem crossing. Also, were this to be a viable mechanism then one might reasonably expect to see a similar level of ^3^BPhe in all of the RCs tested, as the mutations investigated had only very small effects on the absorbance spectrum of the RC.

Regarding mechanism 3, the nature of the RC samples used in this study makes it unlikely that the observed ^3^BPhe signal could be due to RC damage. For the bulk of the samples in which this signal was detected, the RC was not removed from the native membrane, providing no opportunity for loss of the native carotenoid during a purification procedure. Many of the mutant RCs studied have had their structures determined to a good resolution by X-ray crystallography and the resulting data show no indication of structural changes around the carotenoid binding site.

Finally, evidence was seen for the presence of this ^3^BPhe state in purified RCs as well as membrane-embedded complexes, which rules out the possibility that the observed state is associated with pigments located in the membrane outside the RC.

In the view of the presented results and discussion therefore, mechanism 1 seems to be most plausible. However, further investigation is necessary to explore the mechanism of ^3^BPhe formation in detail.

## Conclusions

Obtained kinetics and spectra of WT-RCs and mutant RCs with an altered rate of P^+^H_A_^−^ recombination produced new insight into triplet states formed following photoexcitation. In all samples, the triplet state of spheroidenone was observed with lifetime of ~4 µs. Neither the introduction of point mutations around A-branch chromophores nor the isolation procedure affected the shape of the carotenoid triplet absorption spectrum or its lifetime. For most of the samples, a ^3^BPhe state was observed with lifetime of ~40 µs. In RC-membranes, both ^3^Car and ^3^BPhe formation yields depend on the P^+^H_A_^−^ lifetime but in an opposite way. The mechanism of ^3^BPhe formation needs further investigation.

## Appendix

Assuming that the 495-nm minimum in the fast DAS derives only from photobleaching of Car and the 750-nm minimum in both slow and nondecaying DASes derives exclusively from photobleaching of BPhe, the following can be written from Lambert–Beer law:

A1 $$A_{{1;495\, {\text{nm}}}} = \Delta c_{\text{Car}} \cdot \varepsilon_{{{\text{Car}};495\, {\text{nm}}}} \cdot l$$

A2 $$A_{{2;750\, {\text{nm}}}} + A_{{3;750\, {\text{nm}}}} = \Delta c_{\text{BPhe}} \cdot \varepsilon_{{{\text{BPhe}};750\,{\text{nm}}}} \cdot l,$$

where Δ*c*_Car_ and  Δ*c*_BPhe_ are the laser-depleted concentration of Car and BPhe in ground state, respectively, *ɛ* the steady-state molar extinction coefficient, and *l* is the optical path length. Thus, the ratio of triplet formation yields for Car and BPhe is the following:

A5 $$\frac{{\varPhi_{\text{Car}} }}{{\varPhi_{\text{BPhe}} }} = \frac{{\Delta c_{\text{Car}} }}{{\Delta c_{\text{BPhe}} }} = \frac{{A_{{1;495\, {\text{nm}}}} }}{{A_{{2;750\, {\text{nm}}}} + A_{{3;750\, {\text{nm}}}} }} \cdot \frac{{\varepsilon_{{{\text{BPhe}};750\,{\text{nm}}}} }}{{\varepsilon_{{{\text{Car}};495 \,{\text{nm}}}} }}.$$

The ratio of molar extinction coefficients can be obtained from comparison of steady-state absorption spectra of WT- and carotenoid-less (GML point mutation)-purified RCs. The difference between them is the spectrum of the carotenoid itself. It is assumed that at 750 nm only BPhe absorbs and there are two BPhe molecules per RC with the same contribution to absorption. By comparing these spectra (data not shown) one gets the following:

A4 $$\frac{{\varepsilon_{{{\text{BPhe}};750\,{\text{nm}}}} }}{{\varepsilon_{{{\text{Car}};495\,{\text{nm}}}} }} = \frac{{A_{{{\text{BPhe}};750\,{\text{nm}}}} /2}}{{A_{{{\text{Car}};495\,{\text{nm}}}} }} = 1.09.$$

Using ratio of triplet formation yields, one can get percentage contribution of Car and BPhe in the total triplet in RC:

A5 $$\%_{\text{Car}} = \frac{{\frac{{\varPhi_{\text{Car}} }}{{\varPhi_{\text{BPhe}} }}}}{{\frac{{\varPhi_{\text{Car}} }}{{\varPhi_{\text{BPhe}} }} + 1}} \cdot 100\,\% = \frac{{1.09 \cdot A_{{1;495\, {\text{nm}}}} }}{{A_{{2;750\, {\text{nm}}}} + A_{{3;750\, {\text{nm}}}} + 1.09 \cdot A_{{1;495\, {\text{nm}}}} }} \cdot 100\,\%$$

A6 $$\%_{\text{BPhe}} = 100\,\% - \%_{\text{Car}}.$$
